# The Relationship Between Childhood Sexual Abuse and Eating Disorders Among African American Adolescents in the United States

**DOI:** 10.7759/cureus.37949

**Published:** 2023-04-21

**Authors:** Blessing Adanda Chuku, Nkiru J Obi, Chioma J Anats, Oluwatoyin Z Hambolu, Fiyinfoluwa D Aderibigbe, Nsikan N Akpabio, Lilian O Odion-Omonhimin

**Affiliations:** 1 Medicine and Surgery, University of Port Harcourt College of Health Sciences, Port Harcourt, NGA; 2 Public Health, Washington University in St. Louis, St. Louis, USA; 3 Pediatrics, University of Ghana Medical School, Accra, GHA; 4 Medicine, Richmond Gabriel University, Belair Kingstown, VCT; 5 Pediatrics, All Saints University Dominica, Roseau, DMA; 6 Medicine and Surgery, Bingham University Teaching Hospital, Jos, NGA; 7 Medicine and Surgery, University of Benin, Benin, NGA; 8 Clinical Research, Mercury Clinical Research, Inc., Houston, USA

**Keywords:** child abuse, adverse childhood experience, african american, psychiatric disorders, adolescents, eating disorders, childhood sexual abuse

## Abstract

Background: Childhood sexual abuse (CSA) is one of the numerous adverse childhood experiences. CSA involves coercing a child to engage in sexual acts and is especially heinous as children are unable to consent or advocate for themselves. The formative years of a child are very crucial; therefore, the influence of sexual abuse could be irreversible. The development of an eating disorder is one of the identified consequences of sexual abuse. Using African American adolescents as the sample group, we explored the association between sexual abuse and eating disorders.

Methods: A cross-sectional study was done with secondary data from the National Survey of American LifeAdolescent Supplement (NSAL-A), 2001-2004. Multivariable logistic regression was used to determine the association between CSA and eating disorders (anorexia nervosa, bulimia nervosa, and binge eating disorders) while adjusting for weight satisfaction.

Results: In our sample of 824 African American adolescents, one of whom was also of Caribbean descent, 3.5% reported a history of CSA, while 2.2% reported having an eating disorder. Only about 5.6% of those with a history of CSA reported having an eating disorder. However, other psychiatric disorders were noted among those with a history of abuse, notably panic attacks, which were present in 44.8% of CSA survivors. Our study found no significant association between CSA and eating disorders (OR= 1.14, 95% CI (0.06, 6.20)).

Conclusion: While we sought to relate CSA with the development of eating disorders, we noted no direct association between the two but instead found an association between panic attacks and CSA. The mediating effect of other psychiatric disorders on the development of ED in CSA survivors should be further researched. It is imperative that survivors of CSA undergo immediate psychiatric evaluation. Primary care providers of survivors of CSA should maintain a high index of suspicion and screen for mental health disorders in these patients.

## Introduction

Sexual abuse is a global dilemma that has plagued the world for centuries. Childhood sexual abuse (CSA) is one of the numerous adverse childhood experiences (ACEs), including but not limited to physical abuse, neglect, experiencing violence, substance use problems, mental health problems, and having a family member attempt or die by suicide [1,2]. In addition, ACEs have been linked to adverse health outcomes in children and increased risk of engaging in high-risk behaviors [3,4].

The Centers for Disease Control and Prevention (CDC) describes CSA as “the involvement of a child (person less than 18 years old) in sexual activity that violates the laws or social taboos of society, and that the child either does not fully comprehend, does not consent to, is unable to give informed consent to, or is not developmentally prepared for and cannot consent to” [5]. CSA involves inducing or coercing a child to engage in sexual acts, which include fondling, penetration, and exposing a child to other sexual activities [6].

A child's formative years are crucial; therefore, negative experiences such as sexual abuse during this period can lead to irrefutable damage. The longer the sexual abuse, the more negatively it impacts a child's emotional and physical growth and development [7]. Unlike physical abuse, the effects of sexual abuse on a child, although grave, may not be as glaring. About 90% of CSA victims may not present with abnormal physical findings [8].

CSA is especially heinous as a child cannot consent or advocate for themself. Although many children are victims of sexual assault, there is a misperception of the prevalence statistics of child sexual abuse due to decreased reporting [9,10]. About one in four girls and one in 13 boys in the United States experience child sexual abuse during childhood, the CDC also states, and 91% of the time, the perpetrator is known to the child [11]. 

For many years, researchers have honed in on CSA, carrying out retrospective and cross-sectional studies to determine the prevalence as well as the immediate reaction of the survivors [12-16]. The development of eating disorders (ED) is one of the consequences identified with the trauma of sexual abuse [17-20]. In addition, some studies have also proposed ED as maladaptive coping mechanisms for the trauma of child sexual abuse [8,21,22].

ED are severe persistent disturbances in eating behaviors associated with distressing thoughts and emotions that affect about 5% of the population. ED most often affects women between the ages of 12 and 35 [23]. Various studies have linked CSA with some ED such as anorexia nervosa, bulimia nervosa, binge eating disorder, and avoidant restrictive food intake disorder [20,24-28]. Other conditions such as panic attacks, depression, bipolar disorder, substance abuse, generalized anxiety disorder, sleep disturbances, phobias, and dissociation, as well as long-term side effects such as some of the above, but also post-traumatic stress disorder and sexual disorders, have also been associated with CSA [10,18].

This research examines African American adolescents (between the ages of 10 and 19) [29]. This population was chosen because they fall within the age group most often affected by ED [23] and also depict the race disproportionately identified as victims of abuse [30,31].

Therefore, this study aims to explore the association between ED and sexual abuse in African American adolescents, answering the following questions: 1) What is the prevalence of ED among CSA survivors, 2) Is there a relationship between CSA and ED, and 3) Are there other psychiatric comorbidities that may be more prevalent among CSA survivors compared to non-CSA survivors.

## Materials and methods

A cross-sectional study was done with secondary data from the National Survey of American Life Adolescent Supplement (NSAL-A), 2001-2004 [32]. This dataset was utilized due to a lack of recent data examining these issues in adolescents and African Americans. The NSAL-A measured the prevalence, age of occurrence, patterns, progression, and comorbidity of DSM-IV disorders among African American and Caribbean adolescents in the United States. This allowed for the identification of risk factors as well as factors that protect against the development and continuance of these disorders. The idea of the NSAL-A was to lay the foundation for future research to recognize childhood manifestations of mental disorders in adults.

A total of 1,170 African American and Caribbean adolescents between ages 13 and 17 were interviewed using a questionnaire from 2001 to 2004. A prior NSAL study was done on adults to investigate mental health disorders in adult populations. The adolescents included in this study were linked to the NSAL adult households. Eligible adolescents in participating households were randomly selected. Two participants were selected from a household with more than one eligible adolescent. Preferably, the two participants were of opposite genders. We utilized the combined 2001 to 2004 data for our research.

Data on the history of rape, other forms of sexual assaults asides rape, lifetime eating disorders such as anorexia nervosa, bulimia nervosa, and binge eating disorders were included in the NSAL-A studies. 

Measures

Independent Variables

Rape: The questionnaire defined rape as someone either having sexual intercourse with you or penetrating your body with a finger or object when you did not want them to, either by threatening you or using force. The verbatim question asked was: Did this ever happen to you? Response options were “yes,” “no,” “don't know,” and “refusal.”

2) Sexual assault: A direct question, “other than rape, were you ever sexually assaulted or molested,” was asked with the same response options of “yes,” “no,” “don't know,” “or refusal."

*Dependent Variables* 

Adolescent lifetime eating disorder: This was a self-reported lifetime diagnosis of one or more of anorexia nervosa, bulimia nervosa, and binge eating disorder. Possible responses were “Yes” and “No.”

Psychiatric co-morbidities: This was a self-reported lifetime diagnosis of one or more of panic attacks, lifetime major depression, lifetime bipolar disorder, substance abuse, generalized anxiety disorder and lifetime psychiatric disorder. Possible responses were “Yes” and “No.”

Analysis

Data analysis was conducted in R version 4.0.3. A sexual assault (other than rape) variable and a rape variable were combined to create a single variable “sexual abuse,” which was then used as the independent variable. A complete case analysis was carried out, and respondents with any missing response on our variables of interest were excluded from our analysis. Three hundred forty-six respondents were excluded bringing the final sample size to 824 African American participants. A detailed descriptive statistics table was provided comparing the characteristics of the study population, such as sex, race, age, grade, residence in a foster home, weight satisfaction, and history of sexual abuse between adolescents with a history of eating disorders and those without. These were expressed as percentages, and the differences in these characteristics were tested using chi-square tests for categorical variables like sex, grade, weight satisfaction, etc., and a t-test for the continuous variable, age. All comparisons were evaluated using an alpha of .05. As groups differed significantly on weight satisfaction, this variable was used as a covariate in the analysis. The prevalence of other psychiatric comorbidities among CSA and non-CSA survivors were also compared using chi-square tests. A bivariate logistic regression model was performed to examine the association between sexual abuse and eating disorders. A multivariate logistic model was then used to determine the association between sexual abuse and eating disorders, adjusting for weight satisfaction. Odds ratios and their corresponding 95% CI were reported. P-values of <0.05 were considered statistically significant.

## Results

The 824 responders in this review were of African American descent. One participant was of both African American and Caribbean descent. 44.4% of study participants were male and 55.6%, female. The mean age of the responders was 15 years with the high school freshman category comprising the largest number of participants (23.2%). 15.7% of participants reported a history of weight dissatisfaction. 3.5% participants admitted to ever experiencing an episode of CSA while 2.2% participants reported a history of an ED (Table 1).

**Table 1:** Characteristics of study participants (NSAL-A 2004, n = 824)

	Total (N=824)
Age in years (Median , IQR)	15.0, 2.00
Sex	
FEMALE	458 (55.6%)
MALE	366 (44.4%)
Grade	
6TH GRADE	19 (2.3%)
7TH GRADE	63 (7.6%)
8TH GRADE	156 (18.9%)
HIGH SCHOOL FRESHMAN	191 (23.2%)
HIGH SCHOOL JUNIOR	135 (16.4%)
HIGH SCHOOL SENIOR	95 (11.5%)
HIGH SCHOOL SOPHOMORE	159 (19.3%)
POST HIGH SCHOOL	6 (0.7%)
Ever in foster care	
NO	802 (97.3%)
YES	22 (2.7%)
Weight satisfaction	
NO	695 (84.3%)
YES	129 (15.7%)
Eating Disorder	
Eating Disorder Absent	806 (97.8%)
Eating Disorder Present	18 (2.2%)
Ever abused	
Abuse Present	29 (3.5%)
No Abuse	795 (96.5%)

More females were found to have a history of ED compared to males. The male:female ratio for ED in our sample was 1:2. Among those with ED, only 5.6% reported a history of child sexual abuse.

Our analysis found that adolescents who were CSA survivors were 14% more likely to have an eating disorder. However, this association did not achieve statistical significance (Table 2).

**Table 2:** Characteristics of study participants by eating disorder status

	Eating Disorder Absent (N=806)	Eating Disorder Present (N=18)	Total (N=824)	P-value
Age in years				
Median [Min, Max]	15.0 [13.0, 17.0]	14.0 [13.0, 17.0]	15.0 [13.0, 17.0]	
Sex				
FEMALE	446 (55.3%)	12 (66.7%)	458 (55.6%)	0.633
MALE	360 (44.7%)	6 (33.3%)	366 (44.4%)	
Grade				
6TH GRADE	18 (2.2%)	1 (5.6%)	19 (2.3%)	0.807
7TH GRADE	62 (7.7%)	1 (5.6%)	63 (7.6%)	
8TH GRADE	153 (19.0%)	3 (16.7%)	156 (18.9%)	
HIGH SCHOOL FRESHMAN	182 (22.6%)	9 (50.0%)	191 (23.2%)	
HIGH SCHOOL JUNIOR	134 (16.6%)	1 (5.6%)	135 (16.4%)	
HIGH SCHOOL SENIOR	94 (11.7%)	1 (5.6%)	95 (11.5%)	
HIGH SCHOOL SOPHOMORE	157 (19.5%)	2 (11.1%)	159 (19.3%)	
POST HIGH SCHOOL	6 (0.7%)	0 (0%)	6 (0.7%)	
Ever in foster care				
NO	784 (97.3%)	18 (100%)	802 (97.3%)	0.777
YES	22 (2.7%)	0 (0%)	22 (2.7%)	
Weight satisfaction				
NO	686 (85.1%)	9 (50.0%)	695 (84.3%)	<0.001
YES	120 (14.9%)	9 (50.0%)	129 (15.7%)	
Ever abused				
Abuse Present	28 (3.5%)	1 (5.6%)	29 (3.5%)	0.894
No Abuse	778 (96.5%)	17 (94.4%)	795 (96.5%)	

With and without adjustment for sex and weight satisfaction, our analysis showed that the association between CSA and ED was not statistically significant (Table 3).

**Table 3:** Association between childhood sexual abuse and eating disorders _*model adjusted for weight satisfaction_ _♱model adjusted for sex and weight satisfaction_

Unadjusted Odds Ratios			
Predictor		OR (95% CI)	p-value
Sexual Abuse		1.63 (0.09, 8.41)	0.639
No Sexual Abuse		ref	ref

Adjusted Odds Ratios			
Predictor		OR (95% CI)	p-value
*Sexual Abuse		1.14 (0.06, 6.20)	0.899
*No Sexual Abuse		ref	ref
♱Sexual Abuse		1.06 (0.06, 5.95)	0.957
♱No Sexual Abuse		ref	ref

Generally, there was a higher prevalence of psychiatric comorbidities among CSA survivors compared to those without a history of CSA. These psychiatric comorbidities include panic attack, a lifetime history of major depression, lifetime bipolar disorder, substance abuse, generalized anxiety disorder, and lifetime psychiatric disorder. However, a significant difference was noted only in the prevalence of panic attacks among CSA survivors and non-CSA survivors. The prevalence of panic attack was 44.8% among those who experienced CSA and 19.2% among non-CSA survivors (p = 0.002) (Table 4).

**Table 4:** Prevalence of psychiatric comorbidities among CSA survivors (NSAL 2004) _*Lifetime psychiatric disorders include panic, social phobia, agoraphobia without panic, generalized anxiety, post traumatic stress, major depression, dysthymia, bipolar (I, II, Subthreshold counted as one disorder), irritable major depression, drug abuse, drug dependence, alcohol abuse, alcohol dependence, oppositional defiant, conduct, intermittent explosive disorder (excluding conduct and Mania), anorexia, and bulimia; binge is included as specified._

Psychiatric Comorbidity	Total	CSA Survivors	Non-CSA Survivors	CSA vs. Non-CSA
	N = 824	N = 29	N = 795	X2
Panic Attacks	20.1%	44.8%	19.2%	9.848 (p=0.002)
Lifetime Major Depression	6.4%	13.8%	6.2%	1.587 (p= 0.208)
Lifetime Bipolar Disorder	1.8%	3.4%	1.8%	4.445e-30 (p= 1)
Substance Abuse	2.4%	6.9%	2.3%	0.957 (p= 0.328)
Generalized anxiety disorder	0.8%	3.4%	0.8%	0.273 (p= 0.601)
*Lifetime Psychiatric Disorder	30.3%	44.8%	29.8%	2.317 (p= 0.128)

## Discussion

This present study sought to ascertain if CSA survivors are at higher risk of developing ED than adolescents without a history of CSA. It was hypothesized that there would be an appreciable relationship between the two, and thus, the goal of the study was to establish such a relationship, determine if the prevalence of ED was comparable between both groups and suggest possible steps to aid these adolescents.

Contrary to our hypothesis, this study did not find a significant relationship between CSA and the development of ED among black adolescents in the United States. We went further to control for variables such as gender and weight satisfaction, also finding no significant association. We, however, found that the prevalence of psychiatric morbidities is higher in CSA survivors. This group had a higher proportion of those who had experienced panic attacks, lifetime major depression, lifetime bipolar disorder, substance abuse, generalized anxiety, and lifetime psychiatric disorder compared to non-CSA survivors, however of all the psychiatric comorbidities, only panic attacks were statistically significant.

Although several studies found an association between CSA and ED, the absence of a significant association in our study is supported by Kinzl et al. whose findings implied that CSA was not imperative for the development of an eating disorder [33]. An older study conducted on women by Finn et al. found no relationship between the occurrence of ED and sexual abuse history. It further explained that their concurrently high prevalence in females may have influenced clinicians’ belief that they are related [34]. Other studies also stated that CSA and some EDs might commonly occur simultaneously but found minimal overlap between them [35,36]. The lack of an association between CSA and ED in our study may be explained by the small prevalence of sexual abuse among the study participants and an even smaller prevalence of ED in adolescents. This is similar to other research that also reported a low prevalence of ED among blacks in the United States, with AN being particularly lower [37,38]. Additionally, an investigation on the relationship between ED and traumatic events like sexual abuse revealed that although a history of sexual trauma seemed prevalent enough in patients with ED to be clinically relevant, a direct relationship between experiencing sexual abuse and having an eating disorder was not established as ED symptoms preceded the sexual abuse event in a significant proportion of their study participants [39].

Our finding of increased prevalence of psychiatric morbidities among CSA survivors, particularly panic attacks, may be explained by the fact that early childhood traumas result in a variety of psychopathologies which in turn increase the vulnerability to disturbances in eating. This is in keeping with studies [14,15,18] which suggested anxiety symptoms and disorders, including panic, fear, phobias, and especially PTSD, as some of the most common responses to CSA. Furthermore, studies investigating this relationship have propounded that impulsive dysregulated behaviors such as borderline personality pathology, dissociation, impulsive, self-destructive behavior, drug use, and PTSD may be causally related to the development of an eating disorder following sexual abuse [19,28,40].

In opposition to our report, a meta-analysis that examined the relationship between CSA and ED [20] found a reliable association between the two, linking CSA to an increased likelihood of ED symptoms. This finding was also consistent with other studies [24,27]. Some studies went further to suggest CSA as a nonspecific risk factor for bulimia nervosa, indicating that CSA and bulimia nervosa has a stronger association than CSA and restricting anorexia [26,28,41]. Caslini et al. also reported an association between CSA and bulimia nervosa, as well as CSA and binge eating disorder, but found no significant association between CSA and anorexia nervosa [25]. The observed link between CSA and ED may be because survivors use binge eating, purging, or starvation as means of regulating continuing emotional distress following the experience of abuse [42]. It could also be that victims keep an obsessive diet, starve or purge in a bid to have “perfect” bodies so as to feel invulnerable, more powerful, or to recover shattered self-esteem [8]. It is interesting to note that similar to our study, some of these studies also utilized secondary data sets.

Some limitations of this study include the small sample size and the utilization of an older data set due to the lack of current data examining these issues in our population of interest. Also, as the prevalence of sexual abuse in adolescents understudied was low, it may have precluded the detection of an association if there really was one. However, studies like ours investigating psychologically impacting topics like sexual abuse and ED may be influenced by a social desirability bias, where participants subconsciously identify with what they think is acceptable about uncomfortable experiences. Finally, we did not distinguish between the types of ED and forms of sexual abuse, as sub-classifying the variables was not the aim of this study and thus could not measure how the different types of EDs can be affected by CSA.

## Conclusions

CSA is not mandatory for the occurrence of an ED; nonetheless, it may be a significant risk factor. While we sought to relate CSA with the development of ED, we instead found an association between panic attacks and CSA.

Since there is a heterogeneity of results in studies on the association between CSA and ED, further research would be required to investigate the role of other factors that may act as mediators to explain the association or lack thereof. In addition, further investigation is required to examine what processes predispose to and protect against the development of psychiatric problems among survivors of CSA. It is imperative that psychiatric evaluation be carried out immediately among survivors of CSA. Primary care providers of survivors of CSA should maintain a high index of suspicion and screen for mental health disorders in these patients as early identification of mental health consequences of sexual abuse and prompt intervention may mitigate the psychological damage that may ensue as a result of CSA.
